# Progress in MOFs and MOFs-Integrated MXenes as Electrode Modifiers for Energy Storage and Electrochemical Sensing Applications

**DOI:** 10.3390/molecules29225373

**Published:** 2024-11-14

**Authors:** Sanjeevamuthu Suganthi, Khursheed Ahmad, Tae Hwan Oh

**Affiliations:** School of Chemical Engineering, Yeungnam University, 280 Daehak-Ro, Gyeongsan 38541, Republic of Korea

**Keywords:** MOFs, MXenes, composites, electrochemistry, energy storage, sensors

## Abstract

The global energy demand and environmental pollution are the two major challenges of the present scenario. Recently, researchers focused on the preparation and investigation of catalysts for their capacitive properties for energy storage devices. Thus, supercapacitors have received extensive interest from researchers due to their promising energy storage features and decent cyclic stability/performance. The performance of the supercapacitors are significantly influenced by the physicochemical properties of the electrocatalyst. In this review article, we have compiled the previous reports on the fabrication of MOFs-based composite materials with MXenes for energy storage and electrochemical sensing applications. The metallic and bimetallic MOFs and MOFs/MXenes materials for supercapacitor applications are reviewed. In addition, MOFs/MXenes-based hybrid composites are also compiled towards the determination of various toxic/hazardous materials, such as metal ions like copper ions, mercury ions, and picric acid. We believe that present review article may benefit the researchers working on the preparation of MOFs-based catalysts for supercapacitor and electrochemical sensing applications.

## 1. Introduction

In recent years, it has been observed that energy crisis and environmental pollution are two major problems for today’s world [1]. It is believed that they may worsen in the future if significant attention is not given to such challenges. The global energy crisis is a pressing issue which may significantly influence people’s lifestyle [2,3]. It is necessary to focus on the design and utilization of energy storage or energy conversions devices [4]. It is expected that developing highly efficient energy storage and conversion systems may emerge as a key solution to address such energy-related issues [5,6]. In connection to this, supercapacitors have received tremendous attention from scientific scholars [7,8,9]. The performance of supercapacitors are significantly influenced by the physiochemical features of electrocatalysts (also known as electrode materials) [10]. Thus, it is very important to design and explore the highly efficient electrode materials for energy storage devices and applications. Previously, various electrode materials, such as MOFs [11,12], metal sulfides [13,14], MXenes [15,16], quantum dots [17,18], covalent organic frameworks [19,20], and two-dimensional (2D) materials [21,22] have been reported for the construction of supercapacitors. The MOFs have demonstrated significant potential as candidates for supercapacitors due to high specific surface areas and organized porous structures [23,24,25]. These crystalline materials are composed of metal ions or clusters coordinated with organic ligands, forming porous frameworks with expansive surface areas [11]. The MOFs can be prepared by utilizing various protocols, such as microwave-assisted, solvo-thermal, hydrothermal, electrochemical, sono-chemical, and mechano-chemical methods. Each method may offer distinct advantages for fine-tuning the physicochemical properties of the prepared MOFs. Ongoing research in MOF synthesis and applications holds great potential for advancements across multiple technological fields [26,27]. The MOFs may be well-suited for use as electrode modifiers for the construction of supercapacitors due to their impressive high specific surface area, abundance of pseudo-capacitive redox centers, and tunable pore size [28]. However, the poor conductive nature of the MOFs is a major challenge for the scientific community.

Similar to MOFs, MXenes (M_a+1_X_a_T_n_, where a = 1, 2, or 3; M represents transition metals like Mo, Ti, Cr, Nb, Sc, and V; X denotes carbon, nitrogen, or both; and T_n_ includes surface terminations such as –Cl, –OH, –O, –F, etc.) have drawn substantial research interest in energy storage applications. This attention is due to their outstanding metallic conductivity, excellent hydrophilicity, distinctive layered structure, and rich surface-functional groups [29,30,31]. However, MXenes have relatively low thermodynamic and environmental stability compared to the MOFs [32]. Thus, researchers designed and combined the properties of the MOFs with MXenes to enhance the electrical conductivity of the MOFs and stability of the MXenes [33]. On the other hand, various toxic materials, metal ions, and compounds such as such as copper (Cu^2+^), lead (Pb^2+^), arsenic (As^3+^), nitrite, benomyl, atrazine, phenol, chloramphenicol, picloram, methamidophos, tyrosine, mycophenolic acid, hygromycin B, and hydrogen peroxide (H_2_O_2_) are widely used in various applications [34,35,36,37]. Such compounds have negative impacts on the environment and human beings, and the monitoring of such materials is necessary [37]. In the previous decade, a large number of MOFs-based electrode materials were prepared and explored for energy storage and electrochemical sensing applications due to their excellent catalytic properties [38,39,40,41,42,43,44]. MOFs materials are promising electrode materials but suffer from low conductivity which need significant attentions [45,46,47,48]. Various methods have been developed for the synthesis of MOFs, but electrical conductivity of the MOFs remains a challenge [49,50,51,52,53]. MXene has excellent conductivity and decent catalytic behavior for electrochemical applications [54,55,56,57,58]. In this regard, MXene can be integrated with MOFs to improve the conductivity and electrochemical performance of the MOFs/MXene-based composite materials.

In this review, we have summarized recent advancements in the development of MOFs and MOF/MXene composites for the construction of supercapacitors and electrochemical sensing of various toxic substances and compounds. We believe that this article will serve as a valuable resource for researchers focused on advancing MOFs, MXene, and MOFs/MXenes-composite-based electrode materials for applications in supercapacitors and electrochemical sensing.

## 2. MOFs as Supercapacitor Material

The MOFs-based electrode materials have been utilized in various combinations. In this section, we have categorized MOFs as pristine MOFs and MOFs/MXenes.

### 2.1. Pristine MOFs Based Supercapacitors

**Figure 1:**
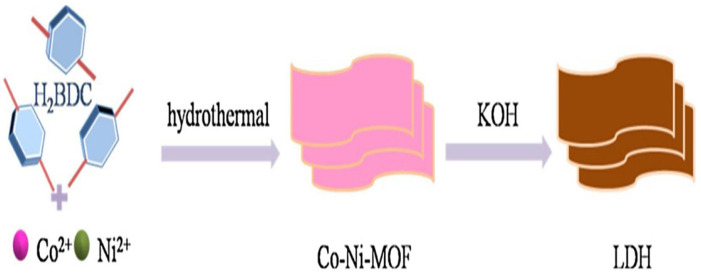
Schematic diagram of the preparation of Co-Ni-MOF-derived LDH. Reproduced with permission [62].

In previous years, various metal-based MOFs, such as zinc (Zn), nickel (Ni), copper (Cu), cobalt (Co), and manganese (Mn), have been synthesized, and their potential for energy storage devices were evaluated by various research groups. In this connection, Farea et al. [59] reported the fabrication of a Zn-based MOF using trimesic as a ligand via a hydrothermal method. Authors characterized the synthesized Zn–MOF by employing Raman spectroscopy, ultraviolet–visible (UV–vis), and photo-luminescence (PL) spectroscopic studies. The synthesized Zn–MOF was further applied as an electrocatalyst for the construction of supercapacitors. The Zn–MOF catalyst exhibited specific capacitance of 58.6 F/g at 0.15 A/g. The Zn–MOFs-modified electrode also showed acceptable stability and retained 82.5% capacitance after 1000 charge–discharge cycles at 0.45 A/g. Hong et al. [60] synthesized Zn–MOF using novel strategies and ligands. In the study, the authors prepared hierarchical porous carbon (HPC) materials by using direct carbonization of Zn–MOFs in the absence of other carbon sources. The authors used Zn-containing MOFs precursors ([Zn_4_O(bdc)_3_], [Zn_2_(bdc)(L-lac)(DMF)], [Zn_2_(bdc)_2_(dabco)], and [Zn_3_(fumarate)_3_(DMF)_2_] (bdc = 1,4-benzenedicarboxylate; dabco = 1,4-diazabicyclo [2.2.2]octane; and L-lac = L-lactate)) to obtain the supercapacitor materials. Authors adopted CV and GCD techniques to evaluate the capacitive features of the proposed electrocatalyst and reported the specific capacitances between 164 and 203 F/g. Salehi et al. [61] reported the synthesis of Zn–MOF using an electrodeposition method. In brief, the authors used zinc (II) nitrate hexahydrate, which was dissolved in N, N-dimethyl-formamide (DMF) and stirred for 20 min. Furthermore, 1,4 dicarboxybenzene (tetraphthalic acid) was separately mixed in ethanol and stored for deposition process. The Zn–MOF film was deposited onto a nickel (Ni) substrate through an electro-deposition process in a two-electrode assembly connected to a DC power source, applying a current density of 30 mA/cm^2^ for 20 min. The resulting Zn–MOF was then analyzed using techniques, including scanning electron microscopy (SEM), X-ray diffraction (XRD), and Fourier-transform infrared spectroscopy (FTIR). Results revealed that Zn–MOF on Ni foam exhibits a layered cuboid structure with uniform dimensions and thickness. The authors expected that such morphology may provide large contact area for electrochemical reactions and shorten the electrolyte pathway. The electrochemical properties of the Zn–MOF-modified Ni foam were evaluated using GCD, CV, and electrochemical impedance spectroscopy (EIS) methods. The obtained results demonstrated a specific capacitance of 288 F/g at 2 A/g and suggested the presence of pseudo-capacitive behavior with a retention cycle of 56% of its initial performance. Cao et al. [62] reported the synthesis of Co-Ni–MOF-derived LDH materials as shown in Figure 1.

**Figure 2:**
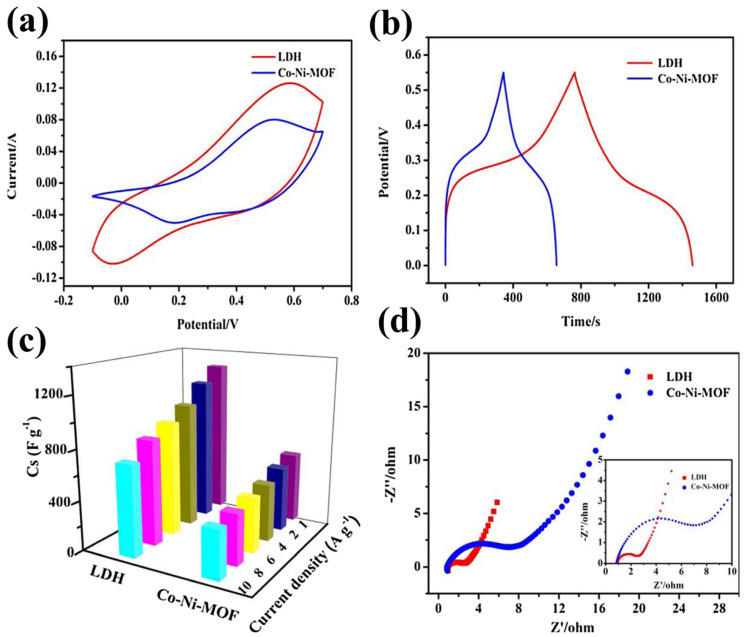
(**a**) CVs, (**b**) GCDs, (**c**) specific capacitance, and (**d**) EIS curves of Co-Ni-MOF and LDH. Reproduced with permission [62].

Furthermore, it was observed that LDH has relatively higher capacitance as observed by CV (Figure 2a). The GCD results also showed that LDH has higher specific capacitance compared to Co-Ni-MOF (Figure 2b,c). The EIS results also showed that LDH has higher conductivity and better charge transfer resistance compared to the Co-Ni-MOF (Figure 2d). This may be ascribed to the better electrocatalytic and conductive properties of the LDH. Du et al. [63] proposed the fabrication of a Cu-based MOF (Cu-MOF) material using a dual-temperature-zone chemical vapor deposition (CVD)-assisted approach. The Cu_3_(HHTP)_2_ (HHTP = 2,3,6,7,10,11-hexahydroxytriphenylene) nanowires arrays were prepared using the proposed CVD method, and the synthesized Cu-MOF was characterized by SEM, XRD, TEM and energy-dispersive X-ray spectroscopic (EDX) techniques.

**Figure 3:**
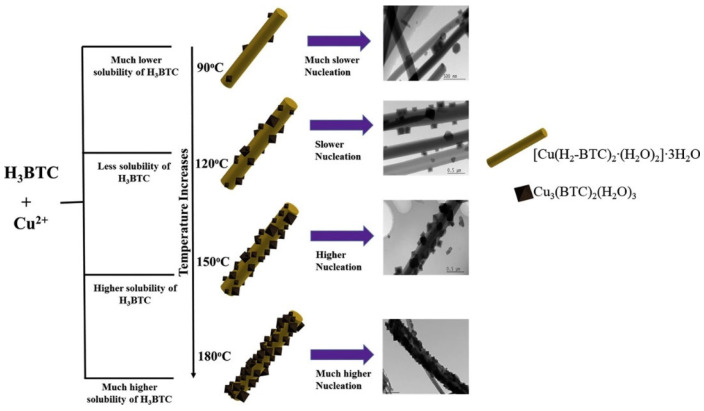
Schematic description for the synthesis of Cu-MOF. Reproduced with permission [68].

The SEM results demonstrated that the obtained Cu-MOF consists of an oriented nanowire-like structure. The obtained results demonstrated a decent specific capacitance of 41.1 mF/cm^2^ at 0.5 A/g. This work expands the synthesis approach from traditional liquid- or gas-phase reactions to a solid–solid reactions, which is anticipated to enable the fabrication of a wide range of conductive MOFs in supercapacitor applications. Liu et al. [64] synthesized Cu-based layered coordination polymer ([Cu(hmt)(tfbdc)(H_2_O)] (where hmt = hexamethylenetetramine, and tfbdc = 2,3,5,6-tetrafluoroterephthalate; Cu-LCP) using a solvothermal method. The Cu-LCP showed higher specific capacitance of 1274 F/g at 1 A/g in a 1 M lithium hydroxide (LiOH)-based system. The Cu-LCP also retained about 88% specific capacitance after 2000 cycles. This may be ascribed to the structural surface properties of the prepared Cu-MOF. The SEM results showed that Cu-MOF comprised of a nanorods-like surface, which may provide better electro transportation. It was believed that the relatively small diameter of the synthesized Cu-LCP nanorods may reduce the ion diffusion length and increase the number of active sites available for electrochemical reactions. Liu et al. [65] proposed the preparation of a conjugated copper catecholate linker (dibenzo-[g,p]chrysene-2,3,6,7,10,11,14,15-octaol (8OH-DBC)=Cu-DBC)-based Cu-MOF using a D_2_-symmetric redox-active ligand in a copper bis(dihydroxy) coordination geometry. The prepared Cu-DBC has decent conductive nature and can be used as an electrocatalyst for energy storage applications. The Cu-DBC was further used as an electrode material, and the obtained results demonstrated a specific capacitance of 479 F/g at 0.2 A/g. The authors also reported an acceptable retention-specific capacitance of 80% after 2000 cycles. Hou et al. [66] proposed the fabrication of intrinsically conductive Cu-MOF nanowire arrays on the self-supported poly-pyrrole membrane for the development of binder-free flexible supercapacitors. The Cu-MOF nanowire arrays may afford decent conductivity with enough active surface area for the accessibility of the liquid electrolyte, while the poly-pyrrole membrane may provide acceptable mechanical flexibility, extra capacitance, and improved charge transfer skeleton. The authors achieved the specific capacitance of 252.1 mF/cm^2^. This research work not only introduces a durable and flexible electrode for supercapacitors which can operate across a wide temperature range but also provides important insights for designing Cu-MOF-based hybrid materials for the construction of supercapacitors. The development of new MOFs are the most promising and important task to further enhance the performance of the supercapacitor electrodes. Ma et al. [67] proposed new Cu-based MOF materials for energy storage applications. The synthesized Cu-MOF (Cu-atrz-BDC) has decent specific surface area of 13.96 m^2^/g and average pore size of 14.6 nm. The Cu-atrz-BDC electrode demonstrated a high specific capacitance of 5525 F/g at 1 A/g. The proposed electrode materials exhibited decent mechanical stability and cyclic stability. Ramachandran et al. [68] reported the synthesis of copper benzene@1, 3, 5-tricarboxylate (Cu@BTC) using a hydrothermal method. The authors found that synthesized Cu@BTC comprises two mixed phases (Cu_3_(BTC)_2_·3H_2_O + {[Cu(BTC-H_2_)_2_·(H_2_O_2_)]·3H_2_O}). The authors also proposed the mechanism for the formation of two phases in the prepared Cu@BTC sample as shown in Figure 3.

**Figure 4:**
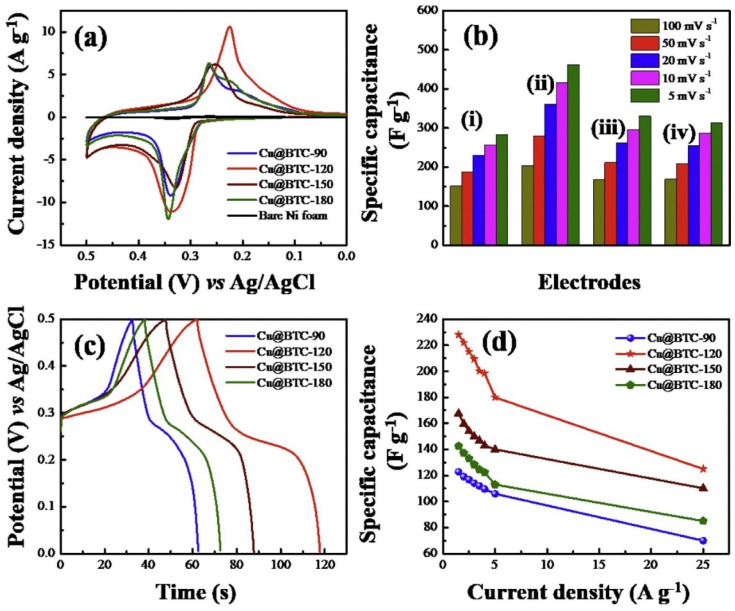
(**a**) CV responses and (**b**) specific capacitance of five different electrodes (i–iv) at different applied scan rates (5–100 mV/s). (**c**) GCD curves at 2 A/g and (**d**) specific capacitance versus current density of different electrodes. Reproduced with permission [68].

The CV responses of the different electrodes (Cu@BTC-120, Cu@BTC-90, Cu@BTC-150, and Cu@BTC-180) were examined (Figure 4a). It was observed that the Cu@BTC-120-based electrode has better CV response compared to the other electrodes. The Cu@BTC-120-based electrode also demonstrates relatively better capacitance (Figure 4b). The GCD curves of the Cu@BTC-90, Cu@BTC-120, Cu@BTC-150, and Cu@BTC-180 electrodes were also obtained as shown in Figure 4c. The specific capacitance versus current density of the different electrodes are shown in Figure 4d. The Cu@BTC-120 electrode demonstrated a significant improvement in specific capacitance compared to other Cu@BTC electrodes, achieving a maximum specific capacitance of 228 F/g at a current density of 1.5 A/g.

**Figure 5:**
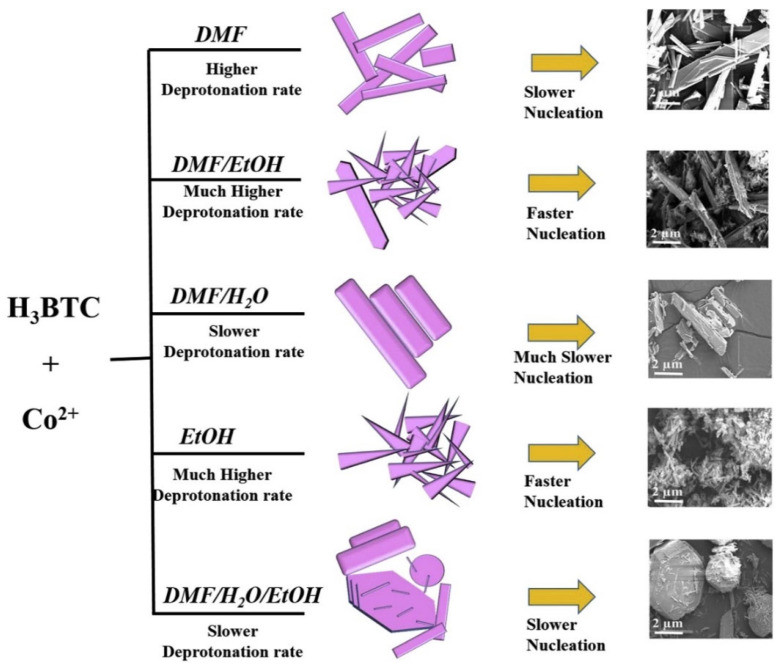
Schematic representation for the preparation of Co-MOFs under different conditions. Reproduced with permission [73].

The Cu@BTC-120 electrode also demonstrates excellent long-term stability, retaining approximately 89.7% of its capacitance after 3000 cycles. The improved performance may be ascribed to the efficient transport of electrolyte ions through the substantial void space between octahedral micro-crystals (Cu_3_(BTC)_2_·3H_2_O) and micro-rods ({[Cu(BTC-H_2_)_2_·(H_2_O_2_)]·3H_2_O}) in the Cu@BTC-120 structure. In other studies, Shaikh and his team introduced Cu-MOF-based electrode materials for energy storage applications [69,70,71]. These materials demonstrated reasonable specific capacitance and power density. While the performance of the Cu-MOF-based materials was satisfactory, the authors incorporated supporting materials like carbon nanotubes, copper oxide, and graphene. Therefore, it is suggested that the improved performance may result from synergistic interactions between the MOF and these supporting materials. Liu et al. [72] synthesized a layered Co-based MOF ({[Co(Hmt)(tfbdc)(H_2_O)_2_]·(H_2_O)_2_}_n_, Co-LMOF, and Hmt = hexamethylenetetramine; H_2_tfbdc = 2,3,5,6-tetrafluoroterephthalic acid) and its (Co-LMOF) physicochemical properties were characterized by using SEM, XRD, and FTIR techniques. The Co-LMOF has particles in nanometer diameter within the range of 35 to 250 nm. The Co-LMOF electrode demonstrated a specific capacitance of 2474 F/g at 1 A/g and retained about 94.3% of its initial capacitance after 2000 cycles. The outstanding electrochemical performance of the proposed electrode material may be attributed to the intrinsic properties of Co−LMOF, ample space for electrolyte storage and diffusion, and the nanoscale size of the particles. Ramachandran et al. [73] described the formation of Co-MOF in different solvents/mixtures by employing solvothermal method. The formation of Co-MOFs under different conditions is described in Figure 5. The Co-MOF (prepared in DMF/ethanol) was further used for supercapacitor application and obtained results showing the specific capacitance of 958.1 F/g at 2 A/g. The reported electrode also retained around 92% capacitance after 3000 cycles.

The study proposed that Co-MOF with a large specific surface area and micro-pore volume may be synthesized by using a DMF/ethanol solvent mixture. It was also proposed that a hybrid structure featuring nano-needles and sharp-edged nanorods may provide more active sites for electrochemical reactions and result in a higher-charge storage capacity compared to other Co-MOFs synthesized in different solvents. In another study, Lee et al. [74] used three dicarboxylic acids with different molecular lengths as organic linkers to tune the surface area and pore-size of the Co-MOFs. It was observed that longer linkers generate larger pores and a greater surface area, while shorter linkers have the opposite effect. In addition, varying molecular sizes of the organic linkers may result in distinct surface architectures of the micro-structure films on the electrode surface. The Co-MOF-based electrodes displayed pseudo-capacitive properties, with the film featuring larger-pores, greater surface-area, and a continuously inter-connected leaflet-like micro-structure with fewer structural interfaces showing superior supercapacitive performance. Thus, of the three Co-MOFs studied, the one with the longer organic linker had larger pores, a greater surface area, and a continuously interconnected leaflet-like microstructure on the electrode surface with fewer structural interfaces, allowing for efficient charge transfer. This Co-MOF electrode exhibited the best specific capacitance of 179.2 F/g. Yang et al. [75] reported the synthesis of the layered Co-MOF, which has a nanosheet-like surface morphology, as revealed by SEM analysis. The authors adopted a simple oil bath synthesis method for the preparation of the Co-MOF. The authors adopted it as an electrode modifier for supercapacitor application and reported an interesting specific capacitance of 2564 F/g at 1 A/g, with acceptable stability of 3000 cycles with 95.9% retention cycles. The authors found that the presence of intrinsic properties, nanosheet-like surface, layered structure, and decent conductivity, of the Co-MOF are responsible for the improved electrochemical performance of the Co-MOF-based supercapacitor. The study suggested that Co-MOF may be a promising electrode material for supercapacitor applications. Lee et al. [76] investigated the pseudo-capacitor behavior of the Co-MOF-based film which demonstrated a specific capacitance of 206.76 F/g. The proposed electrode material exhibited decent stability of 1000 cycles with loss of only 1.5% of its initial performance. Thus, it is clear that Co-MOF are efficient electrode materials with excellent stability. Wang et al. [77] proposed a synthesis of new Co-ordination polymer based Co-MOF material which exhibited high surface area and high nitrogen content. The presence of nitrogen and high surface area may boost the electrochemical performance of the Co-MOF for energy storage applications. The authors used 3 molar potassium hydroxide (KOH) as electrolyte system and examined the supercapacitive properties of the Co-MOF. The Co-MOF-based electrode exhibited specific capacitance of 512 F/g at current density of 1 A/g. The proposed electrode material demonstrated the retention cycles of 97.4% of its initial performance after 40,000 cycles. The excellent performance of the electrode material may be attributed to the electrical properties, large specific surface area, and interconnected pores, which enhance side reactions with the electrolyte and facilitate rapid ion transport between the electrode and the electrolyte interface.

**Figure 6:**
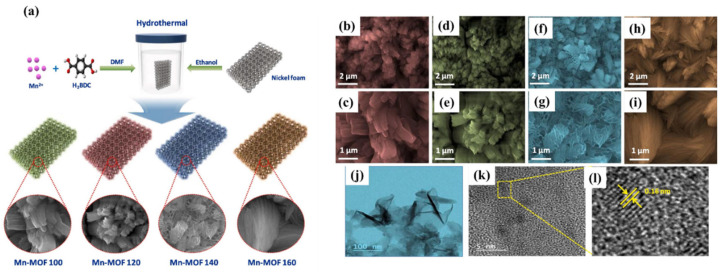
(**a**) Schematic graph for the synthesis of Mn-MOF. SEM images of Mn-MOF synthesized under different temperature conditions: (**b**,**c**) Mn-MOF 100, (**d**,**e**) Mn-MOF 120, (**f**,**g**) Mn-MOF 140, and (**h**,**i**) Mn-MOF 160. TEM image (**j**) and HRTEM images (**k**,**l**) of Mn-MOF 140. Reproduced with permission [81].

Punde et al. [78] have proposed the solvothermal and mechanochemical grinding methods for the preparation of cobalt–benzene-tricarboxylic-acid-based MOF (CoBTC MOF). The comparison between the performance of solvothermal- and mechanochemical-grinding-methods-based CoBTC MOF were examined. The mechanochemical-method-based electrode materials exhibited high specific surface area and improved porosity/diffusion process compared to the solvothermal method. The authors also integrated this MOF with graphene, and the proposed electrode showed specific capacitance of 608.2 F/g at current density of 0.25 A/g in 1 M KOH electrolyte system with capacitance retention of 94.9% after 2000 cycles. Wang et al. [79] reported the benign synthesis of a manganese (Mn)-based MOF for supercapacitor applications. The synthesized Mn-LMOF was further explored as an electrode material, and electrochemical investigations suggested that a high specific capacitance of 1098 F/g and 1178 F/g can be obtained in a 1 M KOH and 1 M LiOH electrolyte system, respectively, at the applied current density of 1 A/g. The authors found that Mn-LMOF has relatively good stability in the LiOH electrolyte compared to the KOH electrolyte and retained 92.6% specific capacitance of its initial performance after 2000 cycles. This acceptable stability and reasonable electrochemical performance of the Mn-LMOF was due to its layered structure and presence of nano-sized particles. Cheng et al. [80] also proposed a novel Mn-based MOF and evaluated its electrochemical activity for supercapacitor applications. In brief, a Mn-MOF (Mn(II)-porphyrins polycondensation polymer (THPP-PA-Mn)) was prepared via esterification of meso-tetra(p-hydroxyphenyl) porphyrin (THPP) with phthalic anhydride (PA) followed by metallizing it with manganese acetate, as shown in Figure 6a. The authors synthesized Mn-MOF at different temperature conditions, and their structural surface properties were examined by using SEM and transmission electron microscopy (TEM). The SEM images of the Mn-MOF 100, (d and e) Mn-MOF 120, (f and g) Mn-MOF 140, and (h and i) Mn-MOF 160 are shown in Figure 6b–i. The TEM and high-resolution TEM images of the Mn-MOF 140 are shown in Figure 6j and Figure 6k,l, respectively. Furthermore, the prepared Mn-MOF was coated on the nickel foam, and its electrochemical performance was determined by using CV and GCD. The observations showed that a specific capacitance of 90.9 F/g at 2.5 A/g with a decay of 13.7% in the specific capacitance after 3000 cycles, which also suggested its good long-term cyclic stability.

**Figure 7:**
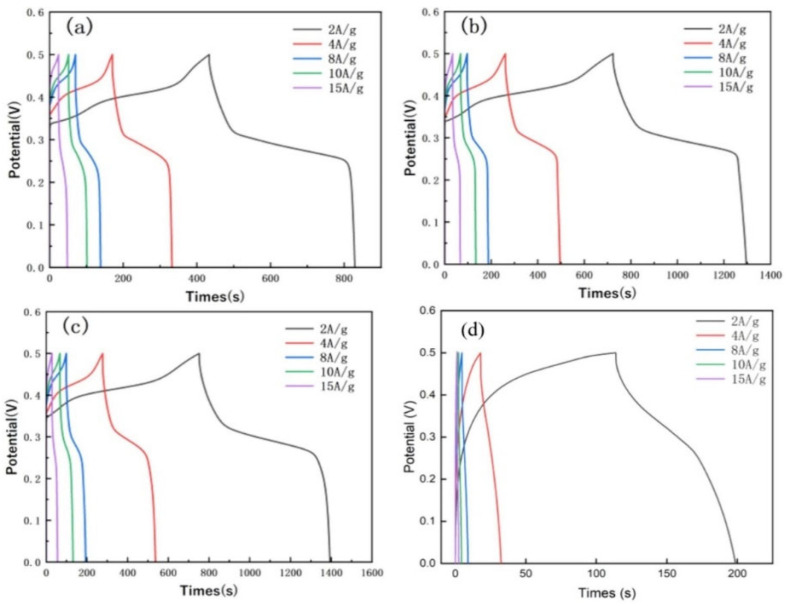
GCD graphs of the (**a**) Ni-MOF (0 mL), (**b**) Ni-MOF (0.5 mL), (**c**) Ni-MOF (1 mL), and (**d**) Ni-MOF (1.5 mL). Reproduced with permission [84].

Shinde et al. [81] reported the hydrothermal synthesis of layered manganese-1,4-benzenedicarboxylic acid-based MOFs [Mn(BDC).nDMF]n (Mn-MOF) for energy storage applications. The Mn-MOF-based electrode exhibited the specific capacitance of 567.5 F/g at 1 A/g. The proposed electrode also retained its initial performance up to 81.80% after 10,000 cycles, which suggested its excellent stability for long-term energy storage applications. Sundriyal et al. [82] synthesized layered manganese-1,4-benzene dicarboxylate (Mn-BDC) (Mn-MOF) using simple strategies and used it as electrode material. The proposed electrode demonstrated specific capacitance of 1590 F/g at 3 A/g. The Mn-MOF-based electrode was also able to retain approximately 82% of its initial specific capacitance after 3000 consecutive GCD cycles. Gao et al. [83] synthesized Ni-MOF using a simple one-step hydrothermal method, with a modified mixed solution of DMF and water instead of pure DMF. The physicochemical characterization revealed that the Ni-MOF forms a loosely stacked layer-cuboid structure with abundant mesopores. Thus, it was believed that it may enhance charge transfer and ion transport. The authors used Ni-MOF as electrode material and obtained a specific capacitance of 804 F/g at 1 A/g with decent stability of 5000 cycles. Liu et al. [84] also explored Ni-MOF as suitable electrode material for energy storage applications. The authors recorded GCD graphs of the Ni-MOF (0 mL), Ni-MOF (0.5 mL), Ni-MOF (1 mL), and Ni-MOF (1.5 mL) at different current densities, as shown in Figure 7a, Figure 7b, Figure 7c, and Figure 7d, respectively.

**Figure 8:**
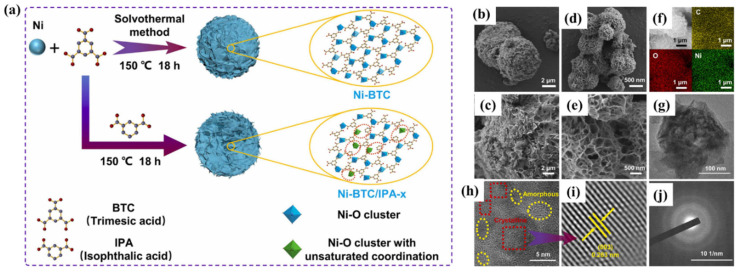
(**a**) Schematic diagram shows the synthesis of Ni-MOF. (**b**) SEM pictures of (**b**,**c**) Ni-BTC and (**d**,**e**) Ni-BTC/IPA-3. (**f**) EDX mapping for C, O and Ni elements, (**g**) TEM, (**h**) HRTEM image, (**i**) crystal faces intensity profile, and (**j**) SAED patterns for Ni-BTC/IPA-3. Reproduced with permission [86].

The Ni-MOF (1 mL); Ni-MOF (0.5 mL); Ni-MOF (0 mL); and Ni-MOF (1.5 mL) exhibited the specific capacitance of 2567.23 F/g; 2291.1 F/g; 1581.1 F/g; and 58.2651 F/g, respectively. The obtained showed that Ni-MOF (1 mL) demonstrated a high specific capacitance of 2567.23 F/g, which was higher than other materials. Sheberla et al. [85] proposed a Ni-based MOF (Ni_3_(2,3,6,7,10,11-hexaiminotriphenylene)_2_ (Ni_3_(HITP)_2_) high electrical conductivity that can serve as the sole electrode material in supercapacitor applications. The proposed Ni-MOF can be used as a binder-free electrode material, and electrochemical investigations revealed that Ni-MOF-based electrodes can bear stability up to 10,000 cycles. Zhang et al. [86] designed and synthesized Ni-based MOF (Ni-BTC (BTC = trimesic acid). The schematic diagram of the synthesis of Ni-MOF has been presented in Figure 8a. The surface morphological features of the Ni-BTC and Ni-BTC/IPA-3 were checked by using SEM, TEM, HRTEM, and SAED patterns, as shown in Figure 8b–j. The SEM and TEM images showed that Ni-BTC/IPA-3 consists of a nanosheets-like surface morphology.

**Figure 9:**
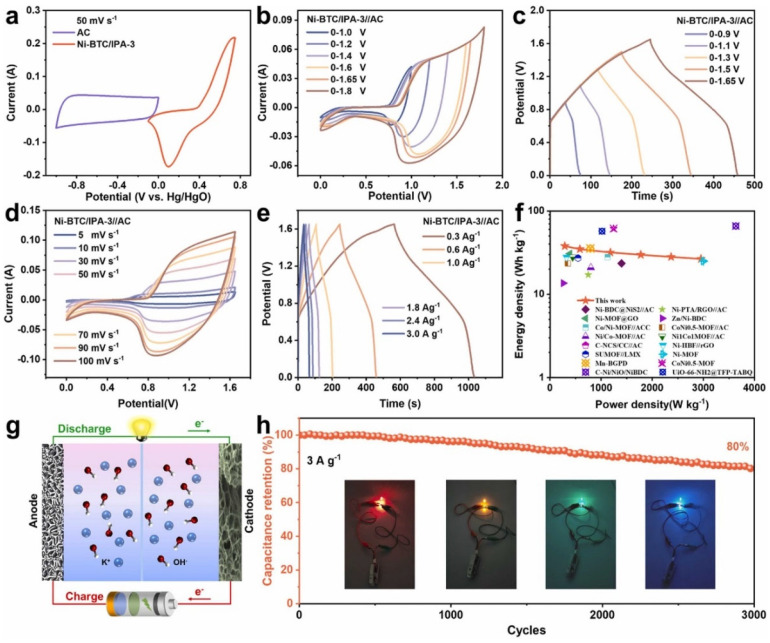
(**a**) CV curves of Ni-BTC/IPA-3 and AC electrode at 50 mV/s. (**b**) CVs at 50 mV/s. (**c**) GCD results at 0.6 A/g at different potential windows, (**d**) CV curves, (**e**) GCD graphs, (**f**) Ragone plots, (**g**) schematic structure of working principle, and (**h**) cyclic performance at 3 A/g. Reproduced with permission [86].

The electrical conductivity and active sites of the Ni-BTC were improved by embedding isophthalic acid, i.e., IPA as missing linkers, resulting in defective Ni-BTC/IPA_-x_. The findings suggest that defects may play a key role in tuning the electronic structure of the metal center, as well as the pore structures and specific surface areas of MOFs, which helps to enhance the performance of supercapacitors. The CV curves of the Ni-BTC/IPA-3 and AC electrode at 50 mV/s are shown in Figure 9a. It can be seen that Ni-BTC/IPA-3 has better electrochemical activity.

The CVs of the Ni-BTC/IPA-3//AC are shown in Figure 9b, whereas the GCD graphs are displayed in Figure 9c under different potential windows. The Ni-BTC/IPA-3//AC exhibited decent electrochemical properties for energy storage devices. The CVs of the Ni-BTC/IPA-3//AC at different applied scan rates are shown in Figure 9d, and the GCD curves are summarized in Figure 9d. The Ragone plot has been presented in Figure 9f. The schematic has been described in Figure 9g. The cyclic performance of the proposed electrode material has been presented in Figure 9g. The Ni-based MOF (Ni-BTC/IPA-3) exhibited specific capacitance of 1209 F/g at 0.5 A/g with a rate performance of 70.8%. Yang et al. [87] also synthesized a layered Ni-based MOF material and characterized it using various sophisticated techniques. The proposed Ni-MOF-based electrode demonstrated decent specific capacitance of 1127 and 668 F/g at 0.5 and 10 A/g, respectively. The proposed electrode also retained good stability of 3000 cycles and held more than 90% capacitance of its initial performance after 3000 cycles. The excellent electrochemical performance of the proposed electrode material was attributed to the intrinsic properties of the Ni-based MOF, particularly its layered structure and favorable exposed facets. Kang et al. [88] reported a new electrode material for supercapacitor applications. The Ni-based MOF (Ni_3_(btc)_2_.12H_2_O) was synthesized by using simple strategies. The authors used KOH as an electrolyte system, and the proposed electrode material (Ni-MOF) demonstrated specific capacitance of 726 F/g and exhibited excellent stability of 1000 cycles with retention cycles of 94.6%. Xu et al. [89] also reported a novel one-dimensional (1D) Ni-based MOF (Ni(HOC_6_H_4_COO)_1.48_(OH)_0.52__1.1H_2_O) using a hydrothermal synthetic method. Ni-MOF has a rods-like surface morphology, which may beneficial for supercapacitor application. The synthesized Ni-MOF nanorods have an average length of 900 nm and diameter of 60 nm. The authors found that Ni-MOF-based electrode demonstrated specific capacitance of 1698, 1385, 1278, 1068, 998, and 838 F/g at 1, 2, 3, 5, 7, and 10 A/g, respectively. The excellent electrochemical properties of the Ni-MOF indicated that the synthesized Ni-based MOF nanorods are highly efficient electrode materials for supercapacitor application and hold significant promise for use in the development of high-performance electrochemical energy storage devices. The electrochemical performance of the reported MOFs for energy storage applications is summarized in Table 1.

### 2.2. MOFs/MXenes-Based Supercapacitors

**Figure 10:**
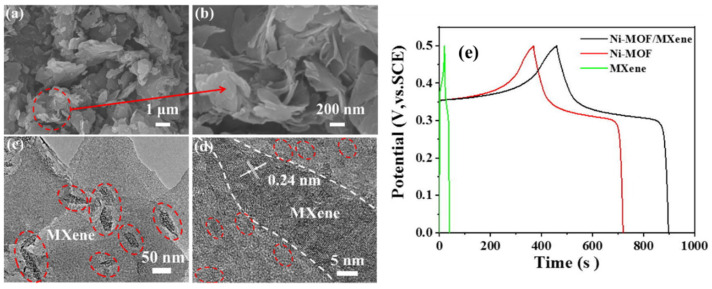
(**a**,**b**) SEM, (**c**) TEM, and (**d**) HRTEM image of Ni-MOF/MXene (red circle shows presence of NiO). (**e**) GCD curve of MXene, Ni-MOF, and Ni-MOF/MXene. Reproduced with permission [94].

It is a well-known factor that MOFs suffer due to their poor electrical conductivity, which really needs significant attention to further enhance the performance of the MOFs-based supercapacitors. It has been observed from the reported literature that integration of MOFs with conductive supporting materials may improve the electrical conductivity of the MOFs materials. In the past few years, MXenes materials with molecular formula of M_n+1_X_n_T_x_ (where T_x_ represents the surface terminations like O, OH, F, and/or Cl that is bonded to the outer M layers) have received extensive interest of the scientific community. The MXenes materials, such as carbon nitride and vanadium carbide, etc., possess decent physicochemical properties, such as high electrical conductivity, and it would be of great significance to integrate MOFs with MXenes as hybrid materials for supercapacitor applications. In connection to this, Yang et al. [90] proposed the synthesis of Ni-MOF integrated V_2_CT_x_ (Tx = –O, –OH and –F surface groups) MXenes composite on Ni foam by using temperature-controlled annealing process. The Ni-MOF/V_2_CT_x_-modified electrode was further used as supercapacitor electrode, which demonstrated specific capacitance of 1103.9 C/g at 1 A/g. The proposed electrode also has acceptable cyclic stability of 15,000 cycles. This stability may be ascribed to the presence of synergism between the Ni-MOF and V_2_CT_x_ material. Zhu et al. [91] also reported the synthesis of a typical Ni-MOF (Ni-HHTP, HHTP = 2, 3, 6, 7, 10, 11-hexahydroxytriphenylene) and prepared its composite with MXene. The synthesized MXene@Ni-HHTP-2 was further explored as an electrode material which showed the specific capacitance of 416.6 F/g at 0.5 A/g. Xie et al. [92] reported that the synthesis of MOF derived Co-Fe oxide porous nanorods integrated with MXene and used it as a supercapacitor material. The proposed material exhibited decent performance in terms of capacitance and stability of 10,000 cycles. Shingte et al. [93] synthesized MOF-derived rhombohedral-shaped nickel ferrite nanoparticles (NFO NPs) into the Ti_3_C_2_T_x_ MXene layers. The 8% NFO NPs-loaded MXene (MXene/NFO-8)-based electrode showed the specific capacitance of 660 F/g at current density of 1 A/g. Qu et al. [94] proposed the synthesis of Ni-MOF/Ti_3_C_2_T_x_ using a sonochemical method. The authors recorded SEM, TEM, and HRTEM images of the synthesized Ni-MOF/Ti_3_C_2_T_x_ to study the morphological features (Figure 10a–d). The authors observed that Ti_3_C_2_T_x_ are uniformly dispersed on the surface of the Ni-MOF, which improved the electronic conductivity and inhibited the aggregation of Ni-MOF nanosheets. The GCD curves of the MXene, Ni-MOF, and Ni-MOF/MXene are displayed in Figure 10e. The Ni-MOF/Ti_3_C_2_T_x_-based electrode showed the specific capacitance of 867.3 F/g at 1 A/g. Compared to the pristine Ni-MOF, the incorporation of Ti_3_C_2_T_X_ improves the electrical conductivity of Ni-MOF nanosheets and effectively prevents their agglomeration, leading to enhanced electrochemical performance.

**Figure 11:**
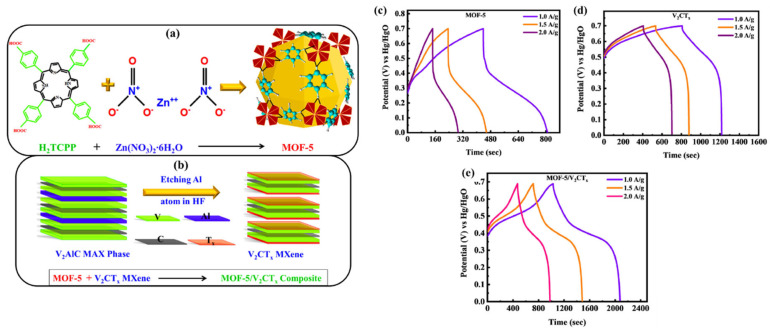
Schematic graph shows the synthesis of (**a**) MOF-5 and (**b**) V_2_CT_x_ MXene and MOF-5/V_2_CT_x_ MXene. GCD curves of the (**c**) MOF-5, (**d**) V_2_CT_x_ MXene, and (**e**) MOF-5/V_2_CT_x_-MXene-based electrodes for supercapacitors. Reproduced with permission [95].

Hassan et al. [95] prepared a novel MOF-5 using a simple hydrothermal approach (Figure 11a), where V_2_CT_x_ MXene was subjected to standard HF etching, followed by the synthesis of a MOF-5/V_2_CT_x_ composite in a one-step hydrothermal process. The MOF-5 and V_2_CT_x_, used as template or precursor sources, have some limitations, such as inconsistent pore size distribution in the carbonized product, durability issues, poor performance, and challenges in cost-effective synthesis. Therefore, authors used V_2_CT_x_ MXene along with affordable conductive linkers for synthesizing MOF-5/V_2_CT_x_ composites, which may offer an alternative approach to the preparation of versatile electrode materials. The synthesized MOF-5/V_2_CT_x_ composite exhibited high porosity, numerous active sites, and uniform dispersion of the MOF-5 porous structure on the V_2_CT_x_ MXene, which makes it a promising electrode materials for energy storage applications.

**Figure 12:**
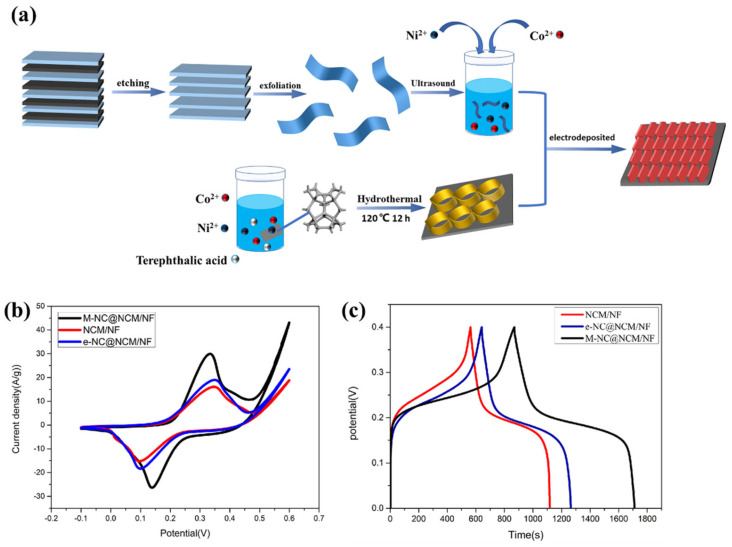
(**a**) Schematic graph shows the synthesis of M-NC@NCM/NF electrode. (**b**) CV and (**c**) GCD curves of the different electrodes. Reproduced with permission [98].

The CD curves of the MOF-5-, V_2_CT_x_ MXene-, and MOF-5/V_2_CT_x_-MXene-based electrodes are shown in Figure 11c, Figure 11d, and Figure 11e, respectively. The synthesized MOF-5/V_2_CT_x_-based electrode demonstrated specific capacitance of 961 C/g in the three-electrode system and 235 C/g in the two-electrode system. Liu et al. [96] synthesized three-dimensional (3D) metal carbides, nitrides, and carbonitrides/MOFs composites (Ti_3_C_2_T_x_/Cu-BTC, Ti_3_C_2_T_x_/Fe,Co-PBA, Ti_3_C_2_T_x_/ZIF-8, and Ti_3_C_2_T_x_/ZIF-67) to improve the performance and stability of the supercapacitors. Further, a Ti_3_C_2_T_x_/ZIF-67/CoV_2_O_6_ composite was prepared, which demonstrated specific capacitance of 285.5 F/g. In another work by Li et al. [97], MXene was directly utilized as a titanium (Ti) source to coordinate with an organic ligand, which formed Ti-MOF sheets. To enhance the electrochemical performance, mesopores were introduced during the preparation of 2D Ti-MOF, creating hierarchical porous Ti-MOF@Ti_3_C_2_T_x_ hybrids. The Ti-MOF@Ti_3_C_2_T_x_ exhibited decent electrochemical performance for energy storage applications. Wang et al. [98] adopted hydrothermal method for the synthesis of bimetallic Ni-CO-MOF on Ni foam and integrated with MXene as shown in Figure 12a. Further, MXene-Ni-Co@NiCo-MOF/NF (M-NC@NCM/NF) nanosheets was obtained using electrodeposition method. Due to the redistribution of metal cations between the MXene layers, nanosheets produced through electrodeposition in an electrolyte containing MXene display a distinctive hexagonal nanosheet morphology. The CV and GCD curves of the different electrodes are shown in Figure 12b and Figure 12c, respectively. This unique structure improves electrochemical performance, maintaining a capacity retention rate of 75.3% after 5000 cycles, with a specific capacity reaching 2137.5 F g^−1^ at a current density of 1 A g^−1^.

Shavita et al. [99] reported the synthesis of Ti_3_C_2_T_x_/Ni-MOF composite using solvothermal method. It was believed that interaction between amino groups and MXene may facilitate the electron transfer pathway and enhance the electrochemical performance of the prepared Ti_3_C_2_T_x_/Ni-MOF composite. The synthesized Ti_3_C_2_T_x_/Ni-MOF composite showed an increased surface area, promoting efficient electrolyte ion diffusion, along with enhanced porosity, numerous redox-active sites, and improved electrical conductivity. The Ti_3_C_2_T_x_/Ni-MOF-composite-based electrode demonstrated a specific capacitance of 536 F/g, which was higher than that of the pristine Ni-MOF (114 F/g) or Ti_3_C_2_T_x_ (224 F/g). The enlarged surface area and expanded interlayer spacing between MXene sheets contributed to a higher capacitance of the Ti_3_C_2_T_x_/Ni-MOF. Hassan et al. [100] also reported the synthesis of an Ag-MOF@V_2_CT_x_ composite which showed a specific capacitance of 2180 C/g at 2 A/g. Ali et al. [101] used the hydrothermal method for the preparation of a NiMn-MOF/MXene composite and characterized it using various sophisticated techniques.

**Figure 13:**
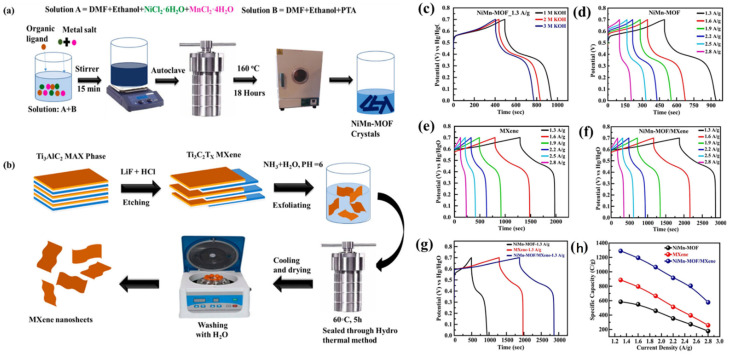
Schematic picture shows the synthesis of (**a**) NiMn-MOF and (**b**) hydrothermal preparation of MXene. GCD curves of Ni-Mn-MOF in different concentrations of KOH (**c**) and at (**d**) different current densities. GCD graphs of the (**e**) MXene, and (**f**) NiMn-MOF/MXene at different current density. GCD curves of the (**g**) NiMn-MOF, MXene, and NiMn-MOF/MXene. (**h**) Specific capacity of NiMn-MOF, MXene, and NiMn-MOF/MXene. Reproduced with permission [101].

**Figure 14:**
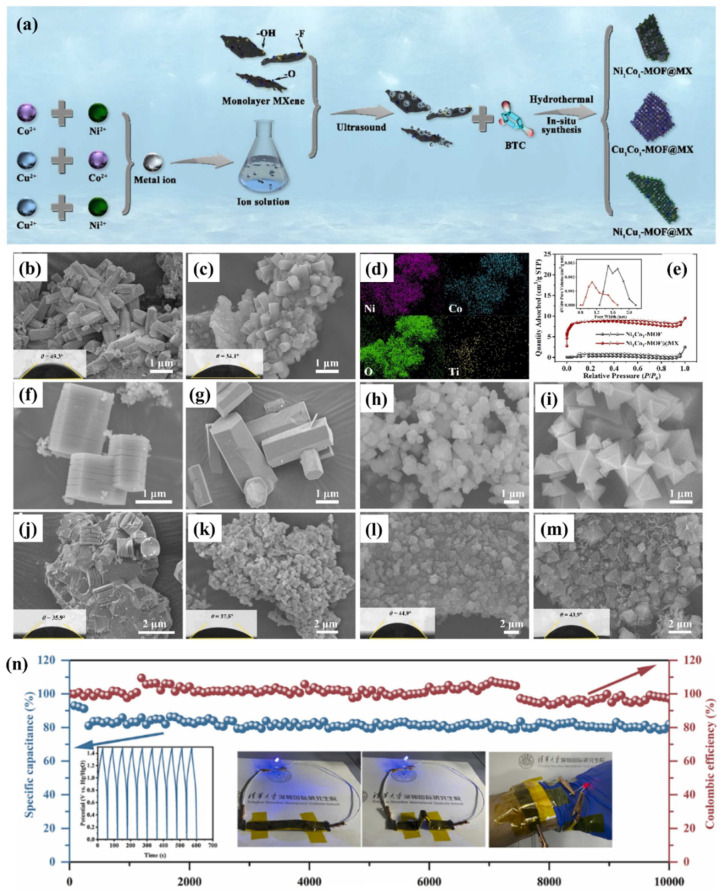
(**a**) Synthetic illustration for the preparation of electrode materials. SEM image and contact angle of (**b**) Ni_1_Co_1_-MOF and (**c**) Ni_1_Co_1_-MOF@MXene. (**d**) EDX and (**e**) BET-BJH curves of Ni_1_Co_1_-MOF@MXene. SEM image of (**f**) Ni_1_Co_3_-MOF, (**j**) Ni_1_Co_3_-MOF@MXene, (**g**) Ni_3_Co_1_-MOF, (**k**) Ni_3_Co_1_-MOF@MXene, (**h**) Cu_1_Co_1_-MOF, (**l**) Cu_1_Co_1_-MOF@MXene, (**i**) Ni_1_Cu_1_-MOF, and (**m**) Ni_1_Cu_1_-MOF@MXene. (**n**) Testing of Ni_1_Co_1_-MOF@MX//AC all-solid-state flexible supercapacitors. Reproduced with permission [102].

The synthesis of NiMn-MOF and hydrothermal treatment for MXene are shown in Figure 13a and Figure 13b, respectively. The GCD graphs of the NiMn-MOF exhibited the high specific capacitance in a 1 M KOH electrolyte compared to the 2 M or 3 M KOH system as shown in Figure 13c. The GCD graphs of the NiMn-MOF at different applied current densities are summarized in Figure 13d. It can be observed that NiMn-MOF has high electrochemical performance at a current density of 1.3 A/g. The MXene-based electrode also exhibited improved specific capacitance compared to the NiMn-MOF at current density of 1.3 A/g (Figure 13e). Further, enhancement in the specific capacitance was also observed for the prepared NiMn-MOF/MXene composite compared to the pristine NiMn-MOF or MXene (Figure 13f–h). The proposed NiMn-MOF/MXene electrode material showed specific capacitance of 215.5 C/g with retention capacity of 90% after 5000 cycles. Ji et al. [102] investigated the synthesis of a range of bimetallic M_x_N_y_-MOF materials (such as Ni_1_Co_1_-MOF, Ni_1_Co_3_-MOF, Ni_3_Co_1_-MOF, Cu_1_Co_1_-MOF, and Ni_1_Cu_1_-MOF) and their composites with MXenes. The synthesis of the electrode materials are summarized in Figure 14a. The surface morphology of the synthesized Ni_1_Co_1_-MOF and (c) Ni_1_Co_1_-MOF@MXene. (d) EDX and (e) BET-BJH curves of Ni_1_Co_1_-MOF@MXene. SEM image of (f) Ni_1_Co_3_-MOF, (j) Ni_1_Co_3_-MOF@MXene, (g) Ni_3_Co_1_-MOF, (k) Ni_3_Co_1_-MOF@MXene, (h) Cu_1_Co_1_-MOF, (l) Cu_1_Co_1_-MOF@MXene, (i) Ni_1_Cu_1_-MOF, and (m) Ni_1_Cu_1_-MOF@MXene were checked by employing SEM. The obtained SEM, EDX, and BET results are displayed in Figure 14b–m. The Ni_1_Cu_1_-MOF/MXenes demonstrated a high specific capacitance of 1493.6 F/g at current density of 1 A/g with 52.7 retention after 5000 cycles (Figure 14n). Wei et al. [103] used hard template method for the preparation of MXene@NiCo-MOF and used it as an electrode material, which demonstrated a high specific capacitance of 2078.1 F/g at current density of 1 A/g.

Zheng et al. [104] reported the synthesis of highly conductive and cyclically stable Ti_3_C_2_ MXene@pillared-layer [Ni(thiophene-2,5-dicarboxylate)(4,40-bipyridine)]_n_ MOF composites (MXene@Ni-MOF). The synthesized material has good capacitive properties and reasonable high specific capacitance of 979 F/g was obtained at current density of 0.5 A/g. Chen et al. [105] reported a synthesis of conductive bismuth-catecholate metal−organic frameworks (Bi(HHTP)) with one-dimensional (1D) channels grown on the surfaces of Ti_3_C_2_T_x_ nanosheets (Ti_3_C_2_T_x_/Bi(HHTP)). The authors found that conductive Bi(HHTP) functions both as spacers to prevent the self-stacking of Ti_3_C_2_T_x_ nanosheets and as an active component, delivering battery-type capacitance. Simultaneously, the Ti_3_C_2_T_x_ serve as a framework for the conductive Bi(HHTP), further boosting the overall specific capacitance of the Ti_3_C_2_T_x_/Bi(HHTP) composite. The specific capacitance of 326 F/g was obtained at current density of 0.5 A/g. Ramachandran et al. [106] described the synthesis of Co-MOF spheres and Co-MOF/Ti_3_C_2_T_x_ on Ni foam which can be used as an innovative binder-free electrode material for supercapacitors. The Co-MOF@Ni and Co-MOF/Ti_3_C_2_T_x_@Ni electrodes achieved maximum capacitance of 2872.5 F/g and 3741 F/g, respectively. It was expected that voids within the Co-MOF structure combined with the excellent conductivity of the layered Ti_3_C_2_T_x_ increased active sites and facilitated rapid ion transport. Guo et al. [107] reported that zeolite-imidazole frameworks (ZIFs) may be the promising materials nut has some limitations such as agglomeration, poor conductivity and low stability. Thus, authors used MXene as substrate, and a Ni-doped ZIF-67 (NiCo-ZIF-7) was anchored. The MXene/NiCo-ZIF-67-based electrode exhibited the specific capacitance of 460 F/g. Yang et al. [108] used Co-BDC/Ti_3_C_2_T_x_ as an electrode material and synthesized it by using thermal treatment. The Co-BDC/Ti_3_C_2_T_x_ based supercapacitor electrode showed specific capacitance of 217.3 C/g. The strong interactions between Co-BDC and Ti_3_C_2_T_x_ are responsible for the better specific capacitance of the proposed electrode. Yang et al. [109] reported the preparation of honeycomb-like MXene@CoSe_2_/Ni_3_Se_4_, and the obtained results showed good electrochemical performance with specific capacity of 283 mAh/g at current density of 1 A/g. The proposed electrode also has excellent 80% retention of its initial specific capacitance after 5000 cycles. A self-powered smart system was developed by Xiang et al. [110] by incorporating flexible solid-state Zn-Co MOFs@MXene supercapacitors and polyacrylamide-BaTiO_3_/NaCl (PAM-BTO/NaCl) organic ionic hydrogel sensors. The authors proposed that Zn-Co MOFs@MXene may be a promising electrode material for energy storage devices. Yue et al. [111] successfully prepared Ni/Co-MOF/thin-layer aminated Ti_3_C_2_T_x_ based electrode material (Ni/Co-MOF@TCT-NH_2_) using simple strategies. The Ni/Co-MOF@TCT-NH_2_ showed a high specific capacitance of 1924 F/g at low current density of 0.5 A/g with excellent cyclic stability. Kshetri et al. [112] designed and synthesized Co-MOF on electroconductive MXene–carbon nanofiber mat (MX-CNF). The Co-MOF@MX-CNF was further used as starting material for the preparation of capacitive-type Co-PC@MX-CNF and battery-type MnO_2_@Co_3_O_4_-PC@MX-CNF functional multi-component electrodes. The proposed work reported a specific capacitance of 426.7 F/g at a current density of 1 A/g. The performance of the reported supercapacitors are summarized in Table 2.

## 3. MOFs and MOFs/MXenes in Electrochemical Sensors

It is well understood that MOFs and their hybrid composite materials have shown excellent performance for electrochemical applications. In the previous years, MOFs and MOFs/MXenes are also widely used as efficient electrode material for electrochemical sensing applications.

**Figure 15:**
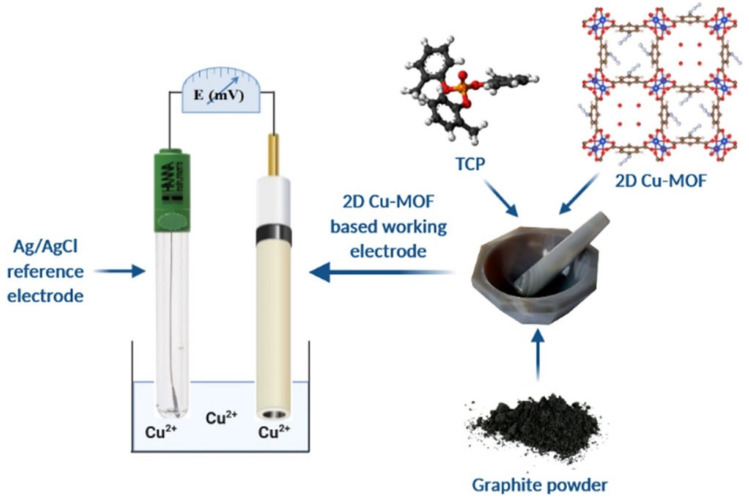
Schematic representation for the preparation of 2D Cu-MOF and working set up for the sensing of Cu^2+^ ions. Reproduced with permission [114].

Zhao et al. [113] reported a novel 2D conductive MOF-based material using 2, 3, 7, 8, 12, 13-hexahydroxyl truxene and copper ions as starting materials. The synthesized c-MOF was further used for electrochemical sensing of paraquat and demonstrated a good limit of detection of 4.1 × 10^−8^ M and linear range of 0.2 to 5 µM. Elashery et al. [114] synthesized 2D Cu-MOF using simple strategies and characterized by sophisticated techniques. The synthetic process for the preparation of 2D Cu-MOF and electrode preparation has been illustrated in Figure 15. The synthesized Cu-MOF was further adopted as electrode material for the sensing of Cu (II) ions and reported decent LOD of 1 × 10^−8^ M.

**Figure 16:**
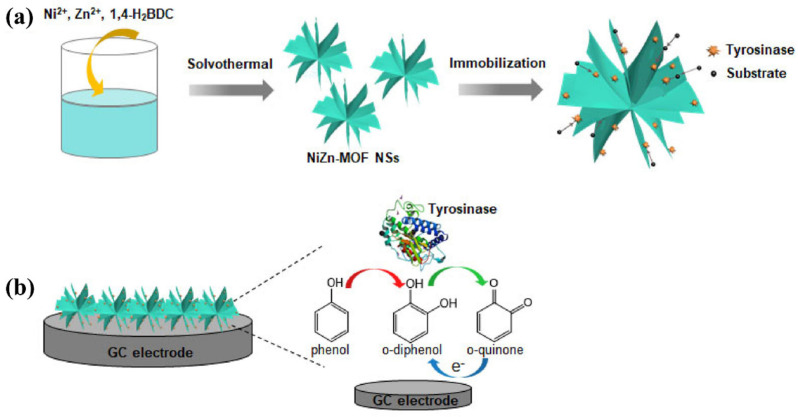
Schematic representation for the preparation of NiZn–MOF (**a**) and working mechanism for the sensing of tyrosinase (**b**). Reproduced with permission [115].

Wen et al. [115] reported the synthesis of 2D bimetallic Ni- and Zn-based MOF nanosheets (NiZn–MOF NSs) with adjustable Ni/Zn ratios which were further utilized as electrode materials for the development of a tyrosinase biosensor (Figure 16a). The synthesized NiZn–MOF was characterized by employing XRD and SEM methods. The XRD pattern exhibited the presence of a decent crystalline nature in the prepared NiZn–MOF whereas SEM results demonstrated that SEM investigations showed that prepared material has flower-like cluster structure. The electrochemical sensing ability of the proposed electrode material was checked by using CV and amperometry methods. The fabricated sensor demonstrated excellent selectivity, long term stability, and high recovery in real samples. The working mechanism for the sensing of tyrosinase has been described in Figure 16b.

**Figure 17:**
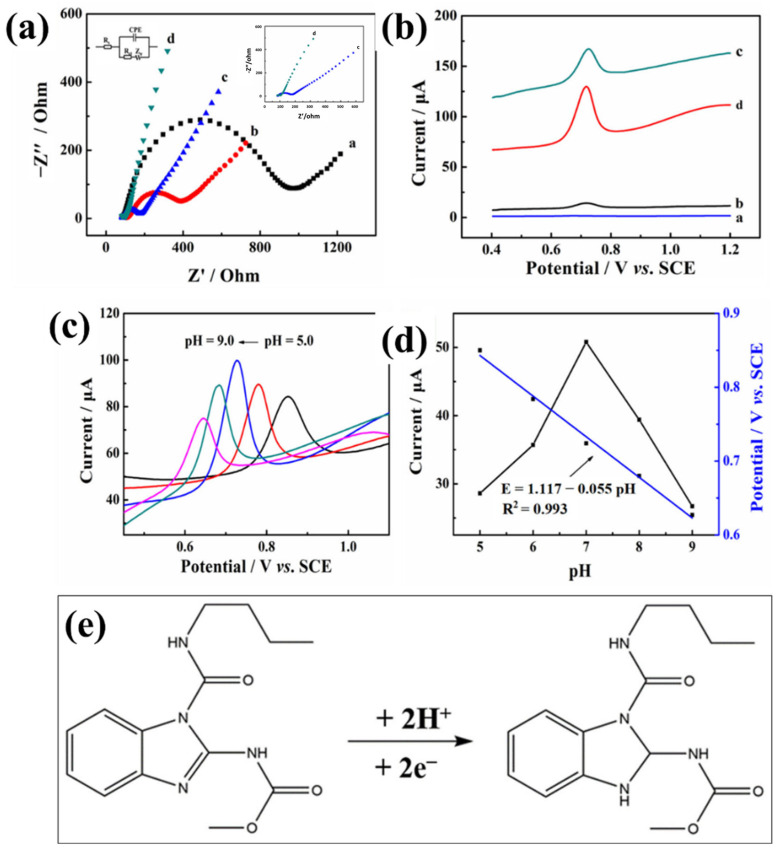
(**a**) EIS of different electrodes (GCE (a), ZIF-L/GCE (b), ERGO/GCE (c), and ERGO@ZIF-L/GCE (d)) in 5 mM K_3_Fe(CN)_6_/K_4_Fe(CN)_6_) system in 0.1 M KCl. (**b**) DPV curves of the different electrodes (GCE (a), ZIF-L/GCE (b), ERGO/GCE (c), and ERGO@ZIF-L/GCE (d)) in 10 µM benomyl. (**c**) DPV curves of the ERGO@ZIF-L/GCE in presence of 10 µM benomyl at different pH values. (**d**) Effects of pH values against current and potential peaks. (**e**) Working mechanism for the sensing of benomyl. Reproduced with permission [117].

Wang et al. [116] synthesized 2D Yb-MOFs for the sensing of picric acid and obtained a LOD of 81.3 nM with linear range of 0.1 to 1 µM. Shi et al. [117] designed and prepared a two-dimensional leaf-like framework-L-embedded electrochemically reduced graphene oxide (ERGO@ZIF-L) and adopted it as an electrode modifier for the electrochemical sensing of benomyl. The authors optimized various parameters, such as material volume, solution pH, and accumulation time, to improve the performance of the proposed electrode for the sensing of benomyl. The Nyquist curves of the different electrodes (GCE (a), ZIF-L/GCE (b), ERGO/GCE (c), and ERGO@ZIF-L/GCE (d)) in 5 mM K_3_Fe(CN)_6_/K_4_Fe(CN)_6_) are shown in Figure 17a. Kit can be seen that ERGO@ZIF-L/GCE has low resistance and high electrical conductivity compared to the other electrodes. The DPV results also showed that ERGO@ZIF-L/GCE has high electrochemical activity and better current response towards the sensing of 10 µM benomyl.

**Figure 18:**
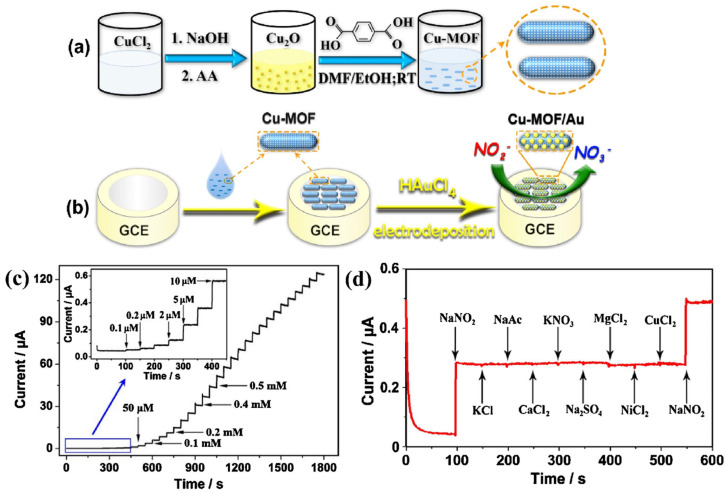
(**a**) Synthetic illustration for Cu-MOF. (**b**) Cu-MOF-based electrode modification illustration. (**c**) Amperometry results for the sensing of nitrite. (**d**) Selectivity results. Reproduced with permission [119].

This obtained results for the ERGO@ZIF-L/GCE was relatively higher compared to the other electrodes, as shown in Figure 17b. The pH of the solution was also optimized, and obtained results showed that ERGO@ZIF-L/GCE has high performance at pH of 7.0 (Figure 17c,d). The proposed sensors exhibited the LOD of 3 nM with linear range of 0.009 to 10.0 µM. In addition, the proposed sensor also demonstrated excellent repeatability, reproducibility, stability, and strong anti-interference performance. The sensing mechanism of benomyl has been described in Figure 17e. The authors believed that improved performance may be attributed to the excellent conductivity, high surface area, and active sites of the prepared electrode material. Du et al. [118] synthesized the glucose oxidase-like Co-MOF for the sensing of atrazine. The Co-MOF was coated on to the electrode surface, and its sensing activity was evaluated for the determination of atrazine. The authors obtained an LOD of 0.65 pM with good selectivity. Chen et al. [119] used Cu-MOF-anchored gold (Au) nanoparticles as electrode material for the determination of nitrite. The authors adopted simple wet chemical method for the synthesis of Cu-MOF, and Au NPs were deposited using an electro-deposition method. The synthesis of the Cu-MOF/Au has been illustrated in Figure 18a. The synthesized Cu-MOF/Au was coated on glassy carbon electrode (GCE) for electrochemical sensing purposes (Figure 18b). The sensing of the nitrite was carried out using an amperometry method. The amperometric results of the Cu-MOF/Au-based electrode for the sensing of nitrite are displayed in Figure 18c. The modified electrode showed an LOD of 82 nM with two linear range of 0.1–4000 and 4000–10,000 μM. The fabricated electrode also exhibited high selectivity (Figure 18d), sensitivity, and strong stability for nitrite detection. The excellent performance may be ascribed to the synergistic interactions, large surface area and porosity of Cu-MOF.

Lu et al. [120] synthesized a 2D nickel phthalocyanine-based MOF nanosheet (2D NiPc-MOF) and used this synthesized MOF as electrode material for the determination of nitrite. The proposed electrode-material-based sensor demonstrated LOD of 2.3 µM with linear range of 0.01 to 11,500 mM. The authors believed that improved performance of the proposed sensor may be ascribed to the presence of high catalytic sites and larger electrochemical active surface area. Wang et al. [121] also proposed a cerium-based MOF (Ce-MOF) on carbon nanotubes (CNTs) substrates and applied it in electrochemical sensing applications. The obtained 2D Ce-MOF featured a distinctive layered structure with hierarchically connected channels, offering highly efficient and stable pathways for the transport of electrons, ions, and mass. The synthesized 2D layered CNTs@Ce-MOF nanosheet hybrid material utilizes Ce as the central ion and electrocatalytically active component for nitrite sensing. A decent LOD of 0.12 µM with linear ranges of 0.65 to 3.25 μM and 3.25 to 7000 μM were reported for the sensing of nitrite. In a report by Bai et al. [122], 2D MOF M-TCPP (M = Cu, Co and Ni) nanofilms and the corresponding 2D MOF M-TCPP nanosheets were prepared in the presence and absence of PVP. Furthermore, Cu-TCPP nanofilm/CNT/GCE was fabricated and explored as the sensing material for the determination of hydrogen peroxide which demonstrated an interesting LOD of 5 nM with linear range of 0.01–3.75 μM and 3.75–377.75 μM. Saraf et al. [123] also investigated the bi-functional property of the Cu-MOF-integrated rGO as electrochemical electrode material for sensing and energy storage applications. The Cu-MOF/rGO-modified GCE demonstrated an excellent LOD of 33 nM with linear range of 3 to 40,000 µM for the determination of nitrite. The versatility of the Cu-MOF/rGO/GCE was evident from its reasonable selectivity in the presence of various interfering species, as well as its capability to detect the nitrite in real samples. Zhang et al. [124] reported a construction of a novel sensor based on a MIL-101(Cr)/XC-72-modified GCE for the sensing of chloramphenicol (CAP). The proposed sensor showed an LOD of 1.5 × 10^−9^ M with acceptable recoveries in real sample. Li et al. [125] fabricated a Zn/Ni-ZIF-8/XC-72/nafion as a sensing material and explored it potential for the determination of lead Pb(II) and Cu (II). The electrochemical performance of the MIL-101(Cr)/XC-72-modified GCE was determined by employing CV, electrochemical impedance spectroscopy (EIS), and differential pulse voltammetry (DPV). The proposed sensor exhibited LODs of 0.0150 and 0.0096 ppm for the determination of Pb (II) and Cu (II), respectively. Hadi et al. [126] described the fabrication and working of a glassy carbon electrode modified with a composite of multi-walled carbon nanotube and Cr-based metal–organic framework (MIL-101, Cr-BDC, BDC = 1,4-benzenedicarboxylate) for the sensing of picloram using square wave voltammetry (SWV) technique. The developed sensor showed an LOD of 0.06 µM and linear range of 0.1 to 12.5 µM. The proposed electrode was successfully applied to determine picloram in tap and river water samples spiked with picloram without requiring any purification step using the standard addition method. The obtained results showed high recovery rates ranging from 97.5% to 105.0%, demonstrating its reliability and accuracy. Song et al. [127] proposed a novel, reliable, and sensitive biosensor using ChE-Chit/MXene/Au NPs/MnO_2_/Mn_3_O_4_/GCE as working electrode. The proposed sensor demonstrated LOD of 1.34 × 10^−13^ M for the sensing of methamidophos. The proposed sensor also exhibited good recoveries in real samples. Huang et al. [128] prepared [MIL-125(Ti)/Fe_x_Ti_y_O_z_]_T_-X (MFTT-X) by using in situ solvothermal and calcination methods. The proposed material was used as a sensing material towards the detection of acetone. Chen et al. [129] proposed the fabrication of a tyrosine sensor using MXene/CNTs/Cu-MOF as an electrode material. The MXene/CNTs/Cu-MOF-based electrode showed an LOD of 0.19 µM with linear range of 0.53 to 232.46 µM. Wang et al. [130] used a molecularly imprinted electrochemical sensor (MIES) modified with Cu-MOF and Ti_3_C_2_T_x_ as sensing platform for the detection of hygromycin B in food and obtained an LOD of 1.92 × 10^−9^ M.

Ge et al. [131] reported the fabrication of Zn-Co MOF/Ti_3_C_2_ MXene/Fe_3_O_4_-MGO/GCE for the sensing of mycophenolic acid and the developed sensor exhibited an LOD of 2.1 × 10^−8^ M for the determination of mycophenolic acid. Xiao et al. [132] synthesized an iron (Fe)-based MOF decorated with MXene (Fe-MOF/MXene) material by using a one-step hydrothermal method.

**Figure 19:**
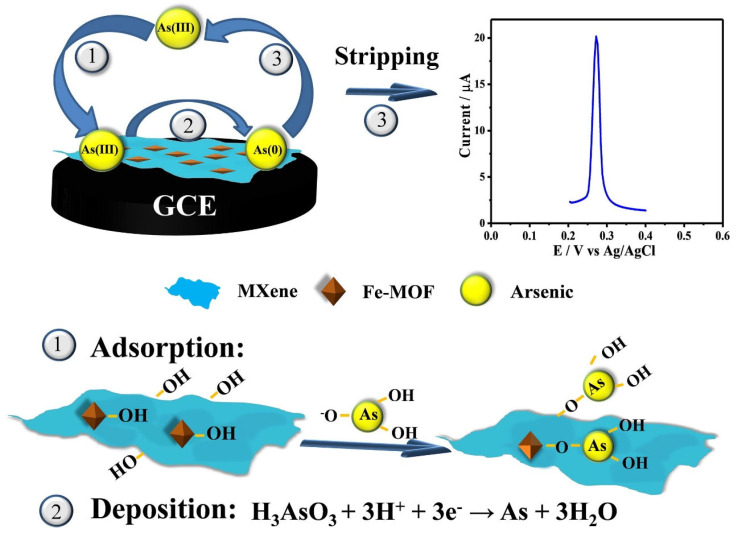
Schematic graph for the determination of As (III) using Fe-MOF/MXene/GCE. Reproduced with permission [132].

The Fe-MOF/MXene was applied as electrode material for the determination of arsenic (As (III)) using CV and square wave anodic stripping voltammetry techniques. The probable mechanism has been illustrated in Figure 19. The low detection limit of 0.58 ng/L was obtained for the sensing of As (III). The analysis of real water samples confirmed that the Fe-MOF/MXene-based sensor is suitable for the practical detection of As (III). The performance of the above-discussed electrochemical sensors are summarized in Table 3.

## 4. Conclusions and Future Perspectives

It is to be summarized that energy demand is increasing with increasing population globally. In this review, we have compiled the previously published articles on the use of MOFs and MOFs/MXenes as electrodes for energy storage and electrochemical sensing of toxic substances such as nitrite. The reported articles showed that MOFs/MXene-based electrode materials have superior performance compared to the pristine MOFs for energy storage and electrochemical sensing applications. However, several challenges remain to be addressed in the development of MOFs/MXenes composites for energy storage and electrochemical sensing applications. These challenges includes refining synthesis techniques for better control of composite morphology and size, exploring new MOF and MXene combinations to improve properties, studying the long-term stability and durability of the composites, and gaining a deeper understanding of the mechanisms driving their electrochemical behavior. Although, various efforts and approaches have been developed to prepare the MOFs/MXene composites. The MOFs/MXene composite demonstrated excellent performance, but still, several challenges, such as toxicity of the HF-etching agent, are the major concern. Thus, green synthetic procedures should be developed to prepare the MXenes and their composite materials.

## Figures and Tables

**Table 1:** Performance of the pristine MOFs-based supercapacitors [59,60,61,62,63,64,65,66,67,68,69,70,71,72,73,74,75,76,77,78,79,80,81,82,83,84,85,86,87,88,89].

Electrode Material	Synthesis Method	Electrolyte	Specific Capacitance (F/g)	Current Density (A/g)	Refs.
Zn-MOF	Hydrothermal	3 M KOH	58.6	0.45	[59]
Zn-MOF	High temperature method	1 M H_2_SO_4_	203	-	[60]
Zn-MOF	Electro-deposition	3 M KOH	288	2	[61]
Co/Ni-MOF	Hydrothermal	2 M KOH	570	1	[62]
Cu-MOF	High temperature assisted stirring	1 M KOH	215	0.5	[63]
Cu-MOF	Solvothermal	1 M LiOH	1274	1	[64]
Cu-MOF	Hydrothermal	1 M KCl	479	0.2	[65]
Cu-MOF	Hydrothermal	3 M NaCl	463 mF/cm^2^	1.25 mA/cm^2^	[66]
Cu-MOF	Hydrothermal	1 M KOH	5525	1	[67]
Cu-MOF	Hydrothermal	3 M KOH	122	1.5	[68]
Cu-MOF	Stirring/slow evaporation	1 M Na_2_SO_4_	244.2	0.8	[69]
Cu-MOF	Layering method	1 M Na_2_SO_4_	685.33	1.6	[70]
Cu-MOF	Slow evaporation	1 M Na_2_SO_4_	380	1.6	[71]
Co-MOF	Mixing method	1 M KOH	2474	1	[72]
Co-MOF	Solvothermal	3 M KOH	958.1	2	[73]
Co-MOF	Solvothermal	0.5 M LiOH	179.2	-	[74]
Co-MOF	Stirring at high temperature	5 M KOH	2564	1	[75]
Co-MOF	Solvothermal	1 M LiOH	206.76	0.61	[76]
Co-MOF	Solvothermal	3 M KOH	512	1	[77]
Co-MOF	Simple grinding method	1 M KOH	608.2	0.25	[78]
Mn-MOF	Solvothermal	1 M LiOH	1098	1	[79]
Mn-MOF	Solvothermal	1 M KOH	109.3	0.25	[80]
Mn-MOF	Hydrothermal	2 M KOH	211.37	1	[81]
Mn-MOF	Solvothermal	1 M Na_2_SO_4_	1590	3	[82]
Ni-MOF	Hydrothermal	2 M KOH	804	1	[83]
Ni-MOF	Hydrothermal	6 M KOH	2567.2	1	[84]
Ni-MOF	Hydrothermal	1 M TEABF_4_/ACN	111	0.05	[85]
Ni-MOF	Solvothermal	3 MKOH	1209	0.5	[86]
Ni-MOF	Hydrothermal	6 M KOH	1127	0.5	[87]
Ni-MOF	Hydrothermal	KOH	726	0.25	[88]
Ni-MOF	Hydrothermal	6 M KOH	1036	1	[89]

**Table 2:** Performance of the pristine MOFs/MXenes-based supercapacitors.

Electrode Material	Synthesis Method	Electrolyte	Specific Capacitance (F/g)	Current Density (A/g)	Refs.
Ni-BDC/V_2_CT_x_/NF-300	Solvothermal	1 M KOH	1103.9 C/g	1	[90]
MXene@Ni-HHTP	Sonochemical	3 M KOH	416.6	0.5	[91]
Co-Fe oxide/Ti_3_C_2_T_X_	Hydrothermal	1 M LiCl	356.4 mF cm^2^	-	[92]
MOF-NFO/MXene	Solvothermal	PVA/KOH	660	1	[93]
Ni-MOF/MXene	Sonochemical	2 M KOH	867.3	1	[94]
MOF-5/V_2_CT_x_	Hydrothermal	3 M KOH	961 C/g	2	[95]
Ti_3_C_2_T_x_/ZIF-67/CoV_2_O_6_	Versatile method	3 M KOH	285.5	1	[96]
HP-Ti-MOF@Ti_3_C_2_T_X_	Soft template	1 M KOH	154	0.2	[97]
MXene@NiCo-MOF	Sonochemical	PVA/KOH	2078.1	1	[98]
Ti_3_C_2_T_x_/Ni-MOF	Solvothermal	1 M H_2_SO_4_	139.4	1	[99]
Ag-MOF@V_2_CT_x_	Hydrothermal	1 M KOH	3114	2	[100]
NiMn-MOF@ MXene	Ultrasonication	1 M H_2_SO_4_	1289.7 C/g	1.3	[101]
Ni_1_Co_1-_MOF@MXene	Ultrasonication	PVA/KOH	1493.6	1	[102]
MXene@NiCo-MOF	Hydrothermal	PVA/KOH	2078.1	1	[103]
MXene@Ni-MOF	Stirring	3 M KOH	979	0.5	[104]
Ti_3_C_2_T_x_Bi(HHTP)	Hydrothermal	3 M KOH	326	0.5	[105]
Co-MOF/Ti_3_C_2_T_x_@NF	Hydrothermal	3 M KOH	3741	3 mA/cm^2^	[106]
MXene/NiCoZDH	Hydrothermal	6 M KOH	877	1	[107]
Co-BDC/Ti_3_C_2_T_x_-300	Solvothermal	1 M KOH	1453 C/g	2 A/m^2^	[108]

**Table 3:** Electrochemical sensing performance of the various reported sensors.

Electrode Material	Technique	LOD	Linear Range (µM)	Analyte	Real Sample	References
2D Cu-MOF	potentiometric	1 × 10^−11^ M	1.0 × 10^−11^ to 1.0 × 10^−9^ M	Cu^2+^	Mate Tea, Tap water	[114]
NiZn–MOF NSs	Amperometry	6.5 nM	1 to 19	Phenol	Water sample	[115]
ERGO@ZIF-L	DPV	3 nM	0.009 to 10.0	Benomyl	Tomato	[117]
Co-MOF	DPV	0.65 pM	1 pM to 100 nM	Atrazine	-	[118]
Cu-MOF/Au	Amperometry	0.082	0.1–4000	Nitrite	River water	[119]
NiPc-MOFs	Amperometry	2.3	0.01 mM to 11,500 mM	Nitrite	Tap water	[120]
2D CNTs@Ce-MOF	DPV	0.12	3.25 to 7000 μM	Nitrite	-	[121]
Cu-TCPP nanofilm/CNT	Amperometry	5 nM	0.01 to 3.75 and 3.75 to 377.75	H_2_O_2_	-	[122]
Cu-MOF/rGO	Amperometry	33	3 to 40,000	Nitrite	Pond water	[123]
MIL-101(Cr)/XC-72	DPV	1.5 nM	1.0 × 10^−8^ to 2.0 × 10^−5^ M	Chloramphenicol	Honey, Eye drops	[124]
Zn/Ni-ZIF-8/XC-72/Nafion	DPV	0.0150 ppm	0.794 to 39.6 ppm	Pb^2+^	Honey, Lake water	[125]
Zn/Ni-ZIF-8/XC-72/Nafion	DPV	0.0096	0.397 to 19.9 ppm	Cu^2+^	-	[125]
Cr-MOF	Voltammetric	0.06	0.1–12.5	Picloram	River water	[126]
AChE-Chit/MXene/Au NPs/MnO_2_/Mn_3_O_4_	DPV	1.34 × 10^−13^ M	10^−12^ to 10^−6^ M	Methamidophos	Fruit	[127]
MXene/CNTs/Cu-MOF	DPV	0.19	0.53 to 232.46	Tyrosine	Human serum	[129]
Cu-MOF/Ti_3_C_2_T_x_	CV	1.92 × 10^−9^ M	5 × 10^−9^ to 5 × 10^−6^ M	Hygromycin B	Fish, Chicken	[130]
Zn-Co MOF/Ti_3_C_2_ MXene/Fe_3_O_4_-MGO	DPV	2.1 × 10^−8^ mol/L	3.21 × 10^−8^ mol/L to 8.9856 × 10^−6^ mol/L	Mycophenolic acid	Silage, maize, alfalfa, wheat	[131]
Fe-MOF/MXene	SWASV	0.58 ng/L	10 to 100 ng/L	As(III)	Lake water, River water	[132]

## Data Availability

No new data were created or analyzed in this study. Data sharing is not applicable to this article.
